# Detection of Microsatellite Instability: State of the Art and Future Applications in Circulating Tumour DNA (ctDNA)

**DOI:** 10.3390/cancers13071491

**Published:** 2021-03-24

**Authors:** Pauline Gilson, Jean-Louis Merlin, Alexandre Harlé

**Affiliations:** Service de Biologie Moléculaire des Tumeurs, Institut de Cancérologie de Lorraine, CNRS UMR 7039 CRAN-Université de Lorraine, 6 avenue de Bourgogne CS 30519, 54519 Vandœuvre-lès-Nancy CEDEX, France; jl.merlin@nancy.unicancer.fr (J.-L.M.); a.harle@nancy.unicancer.fr (A.H.)

**Keywords:** microsatellite instability, cancer, immunotherapy, Lynch syndrome, liquid biopsy, PCR, NGS, droplet digital PCR

## Abstract

**Simple Summary:**

Microsatellite instability (MSI) is a molecular fingerprint for defects in the mismatch repair system (dMMR) and is associated with higher risks of cancers. MSI/dMMR tumours are characterized by the accumulation of mutations throughout the genome, and particularly in microsatellite (MS) DNA repeat sequences. MSI stands as a major biomarker for familial cancer risk assessment, cancer prognosis, and therapeutic choices. Standard-of-care classification of MSI/dMMR tumours is most frequently achieved using immunohistochemistry or PCR-based assay directed against a set of five MS regions. However, novel molecular methods based on tumour tissue or plasma samples have been developed and could enter in the future trends of MSI testing. Here, we provide insights into these emerging approaches and discuss their advantages and limitations.

**Abstract:**

Microsatellite instability (MSI) is a molecular scar resulting from a defective mismatch repair system (dMMR) and associated with various malignancies. MSI tumours are characterized by the accumulation of mutations throughout the genome and particularly clustered in highly repetitive microsatellite (MS) regions. MSI/dMMR status is routinely assessed in solid tumours for the initial screening of Lynch syndrome, the evaluation of cancer prognosis, and treatment decision-making. Currently, pentaplex PCR-based methods and MMR immunohistochemistry on tumour tissue samples are the standard diagnostic methods for MSI/dMMR. Other tissue methods such as next-generation sequencing or real-time PCR-based systems have emerged and represent viable alternatives to standard MSI testing in specific settings. The evolution of the standard molecular techniques has offered the opportunity to extend MSI determination to liquid biopsy based on the analysis of cell-free DNA (cfDNA) in plasma. This review aims at synthetizing the standard and emerging techniques used on tumour tissue samples for MSI/dMMR determination. We also provide insights into the MSI molecular techniques compatible with liquid biopsy and the potential clinical consequences for patients with solid cancers.

## 1. Introduction

The mismatch repair (MMR) machinery is an evolutionarily conserved system responsible for the preservation of DNA homeostasis in cells [1]. The MMR system is composed of the hMutS heterodimers (MSH2/MSH6 and MSH2/MSH3 complexes) that ensure the specific recognition of mispaired nucleotides and small insertion-deletions generated during the replication or recombination processes [2] or resulting from DNA damage [3]. These complexes initiate the repair and recruit the hMutL heterodimers (hMLH1/hPMS2, hMLH1/hPMS1 and hMLH1/hMLH3) to catalyze the mispair excision and error-free resynthesis using the remaining DNA strand as a template for the DNA polymerase [4]. Genetic and epigenetic inactivations of MMR genes cause MMR defects (dMMR) and give rise to a hypermutability phenotype characterized by spontaneous, genome-wide, mutagenesis [4]. This particularly affects the short tandem repeat DNA sequences termed microsatellites (MS) and can predispose to cancer.

According to a recent study conducted on more than 11,000 tissue samples spanning 39 cancer types, microsatellite instability (MSI) was observed within 27 cancer types (3.8% of all cancers analyzed). Twelve cancer types were found with a MSI prevalence greater than 1%, mostly represented by endometrial (31.4%), gastric (19.1%), and colorectal (CRC) adenocarcinomas (16.0%) [5]. Most MSI tumours arise sporadically [6] (74%, 97%, and 63% of endometrial, gastric, and CRC MSI cancer cases, respectively), often associated with an hypermethylation of the MLH1 promoter (all localizations) or a BRAF *V600E* mutation (in CRC specifically) [7]. Others result from inherited cancer predisposition syndromes such as Lynch syndrome (LS). LS originates from a monoallelic germline mutation in one of the four main MMR genes (*MLH1*, *MSH2*, *MSH6*, *PMS2*) or the *EPCAM* gene (3′end deletions of EPCAM gene induce epigenetic silencing of its neighbouring gene MSH2) followed by a somatic inactivation of the second allele [8,9]. LS-related malignancies are predominantly represented by colorectal and endometrial cancers, and less frequently by tumours of the upper urinary tract, small intestine, stomach, biliary system, ovarium, pancreas, brain, and skin [10].

MSI has several implications in the management of patients with solid cancers. First, it can contribute to initial LS screening pipelines as MSI characterizes almost 95% of inherited malignancies associated with LS [10]. LS screening programs have been frequently reviewed and updated. Originally, MSI testing was proposed for patients with solid cancers that meet the Amsterdam criteria or Bethesda guidelines, that include in the latest versions: personal and/or family histories of cancer, development of metachronous or synchronous cancers, young age at onset of cancer within the LS spectrum [11]. Some expert panels now advocate universal MSI testing for all patients with colorectal or endometrial cancers at diagnosis in order to provide better opportunity for cancer prevention in their relatives [12,13,14,15]. MSI status is also of positive prognostic value for localized CRC and gastric cancers. MSI status is commonly correlated with poor likelihood of lymph node invasion and metastases and predicts a favorable outcome in stage II-III diseases [16,17,18,19,20]. The clinical benefit of MSI in resectable tumours seems however reduced when using a treatment with a fluorouracil-based regimen [21,22]. Based on these data, MSI testing is becoming a prerequisite for all stage II CRC and patients with MSI cancers should be spared fluorouracil-based adjuvant chemotherapy according to the National Comprehensive Cancer Network guidelines [17,22,23,24]. In the same manner, MSI typing may guide perioperative chemotherapy decisions in resectable gastric cancers [25]. Finally, MSI has gained considerable attention in recent years because of its predictive role regarding response to immunotherapy across multiple tumour types [26,27]. It is now commonly believed that dMMR/MSI tumours frequently express a high neoantigen burden, making them highly immunogenic and responsive to immune checkpoint inhibitors (ICI) [28,29]. The U.S Food and Drug Administration (FDA) granted approval to nivolumab (a programmed death receptor-1 (PD-1) inhibitor) alone or in combination with ipilimumab (a cytotoxic T-lymphocyte antigen 4 (CTLA-4)-blocking antibody) for patients with metastatic MSI/dMMR CRC [30,31]. Pembrolizumab, another anti-PD-1 agent, also benefited from FDA-approval for agnostic indication in patients with refractory solid cancers [32] or as a frontline treatment for CRC [33], which exhibits MSI/dMMR.

## 2. MSI Standard Reference Testing

Immunohistochemistry (IHC) and PCR-based assays performed on tumour tissue samples (biopsy or surgical resection samples) account for gold standard in determination of MSI/dMMR status (Table 1). IHC shows high sensitivity and specificity in the most frequent LS-associated cancers when exploring the expression of either the 4 main MMR proteins (MLH1, MSH2, MSH6, PMS2) or only MSH6/PMS2 proteins [34,35]. Based on these results, dMMR, as indicated by IHC, leads to reflex testing for LS [36]. Yet, MSI-PCR approaches based on the PCR amplification of MS regions followed by capillary electrophoresis (PCR-CE) have been demonstrated as a reliable alternative to the historical IHC-based testing. Notably, MSI-PCR allows retrieval of cases with preanalytical issues or indeterminate results in IHC as well as IHC false negative results due to rare non-truncating missense mutations in the MMR genes associated with intact antigenicity [37]. The clinical interest of MSI-PCR has particularly increased with the development of pentaplex PCR panels integrating 5 mononucleotide and quasi-monomorphic MS regions (including BAT-25 and BAT-26), which improved the assay sensitivity and obviated the need for analyzing paired normal tissue for MS length comparison [38,39,40]. The Promega^®^ MSI analysis system appears as one of the most investigated commercial PCR assay using 5 mononucleotide markers (*BAT-25*, *BAT-26*, *MONO-27*, *NR-21* and *NR-24*) for MSI typing and 2 pentanucleotide regions (PentaC and PentaD) to detect sample mix-up or contamination [41]. Currently, IHC and MSI-PCR methods are nearly equally proficient in identifying MSI/dMMR colorectal, endometrial, and probably gastric cancers [42,43,44,45,46]. The concordance between the two techniques appears less certain in other cancers and may differ depending on the tumour tissue of origin [47,48]. In practice, these techniques are complementary since IHC identifies the cause of dMMR while PCR explores the consequences at the nucleic acids level on MS length. Because the theranostic implications of MSI are substantial and neither approach is able to detect all MSI/dMMR cases, it has been proposed that both assays should be applied simultaneously or sequentially in order to avoid MSI misinterpretation [36,49,50].

## 3. Other MSI Approaches on Tumour Tissue Samples

Other molecular approaches based on the analysis of tumour tissue samples have recently emerged with the aim of improving sensitivity and specificity compared to conventional MSI/dMMR testing. They may represent present or future valuable options to conventional MSI testing.

### 3.1. Histopathology-Based Approaches

In patients with unresectable or metastatic cancers, biopsy or surgical resection specimens are often difficult to obtain and cytology of body fluids can be the only sample available for diagnosis. In this context, Jacobi et al. evaluated the feasibility to determine MSI status in cytologic material from patients with colorectal or endometrial cancers [52]. IHC staining was performed on cell block sections prepared from cytologic specimens and results were concordant in 85% cases (45/53) with IHC/MSI-PCR results from matched surgical samples. Inconclusive and false-negative cell block results arose in 11% and 4% cases, respectively, resulting from low tumour cell content, staining in cells indefinite for tumour, staining heterogeneity in tumour cells, or lack of internal control staining. In the absence of surgical specimens available, cytologic samples could thus represent a promising source of material for dMMR testing. Particular attention is, however, needed for the interpretation of the results.

Moving into the era of universal MSI/dMMR testing, there is a growing need for faster, easier-to-perform, and more affordable approaches than the conventional methods. Artificial-intelligence methods have been recently proposed to directly predict MSI status from routine haematoxylin and eosin-stained slides, which are routinely available for almost all patients with cancer [53,54,55,56,57]. Each tumour image is segmented into thousands of tiles in which deep learning models assign MSI-score based on different features. For each slide, MSI classification is inferred based on the score of the majority of the tiles [54]. Studies support that machine learning algorithms are useful to predict MSI status, however they require large cohorts for training and validation and are only relevant on cohorts with similar patient and sample characteristics than the training datasets [53]. The utility of such approach for forecasting ICI efficacy is still to be demonstrated.

### 3.2. PCR-Based Assays

Emerging PCR-based assays differ from the standard reference PCR method by the nature and number of targeted MS markers and/or the read-out strategy of PCR products (Table 2). For example, some groups integrated the analysis of novel long mononucleotide repeats (LMR) besides the traditional pentaplex panel [58,59]. Bacher et al. identified a large number of mononucleotide repeats with increased repeat length (40–60 bp) compared to the MS markers traditionally used for MSI testing [59]. They showed that MS mutation rate exponentially increases with the number of repeat units. Thus, the analysis of LMR markers allowed to enhance the detection sensitivity of the MSI-PCR assay. The commercial LMR MSI analysis system (Promega^®^, Madison, WI, USA) that includes four conventional mononucleotide MS markers (*BAT25*, *BAT26*, *Mono27*, *NR21*, and *NR24*) and 4 LMR markers (*BAT-52*, *BAT-56*, *BAT-59* and *BAT-60*) notably reached a higher agreement with IHC in colorectal samples compared to the Promega^®^ MSI analysis system [58]. The utility of such panel still needs to be confirmed for non-colorectal specimens.

Others substituted the capillary electrophoresis (CE) step by either denaturing high-performance liquid chromatography (DHPLC) or high resolution melting (HRM) to detect PCR products from MS markers [60,61,62]. DHPLC has the advantage of enabling high throughput determination of MSI and being exempt from confounding stutter peaks, a frequent artefact observed with PCR-CE and PCR-HRM resulting from DNA slippage during PCR amplification [60,63,64]. The limit of detection for MSI using DHPLC was found as low as 1, mutated out of 100 non-mutated alleles [60].

Others proposed the replacement of the conventional pentaplex panel by a sole MS marker, which has been found highly informative for MSI in CRC. In fact, the high mutation rate observed in MSI tumours seems to preferentially appear in large microsatellite regions, such as the T17 intron repeat of the chaperone Heat Shock Protein 110 (*HSP110*) gene, upstream of the exon 9 splice acceptor site [65]. Somatic deletions in *HSP110 (T17)* were reported in almost all MSI CRC and were associated with the expression of a mutant truncated HSP110 protein conferring better prognosis and sensitivity to chemotherapy [66,67]. In this context, the research of mutations in the *HSP110 (T17)* marker has been proposed as an alternative to conventional MSI testing assays since it provides better sensitivity and equal specificity in CRC [68,69] and requires the analysis of a sole quasi-monomorphic marker (Table 2). Allelic alterations in *HSP110 (T17)* were historically detected using PCR followed by fragment analysis [68,70], but a novel enrichment-based strategy using E-ice-COLD-PCR seems to better detect MSI with a 20-200-fold gain of sensitivity compared to conventional PCR [71]. The analysis of the *HSP110 (T17)* region may represent a promising molecular tool for MSI stratification; however, its utility in non-CRC samples is currently unknown and needs to be investigated before its use in clinical practice [72,73].

The Idylla^®^ MSI assay (Bioartis, NV, Mechelen, Belgium) is a fully-automated PCR-based system that comprises the analysis of seven novel monomorphic homopolymer regions (*ACVR2A*, *BTBD7*, *DIDO1*, *MRE11*, *RYR3*, *SEC31A*, *SULF2*) in a single-use cartridge with all reagents on board (Table 2) [74]. In colorectal, endometrial, and gastric cancers, the Idylla^®^ system provides a high concordance (>96%) and lower failure rate compared to the standard reference methods [75,76,77,78]. Less is known about its performance in other cancers and further studies are needed to confirm its interest in such cases [79]. The Idylla^®^ benefits from a minimal hands-on-time (<5 min), does not require the analysis of matched normal tissues for MSI interpretation, and gives results in only 2.5 h [78]. Moreover, this easy-to-perform system provides an automated interpretation of the MSI status, which is certainly adapted for in-house testing.

We recently evaluated a droplet digital *PCR* (ddPCR)–based assay (Bio-Rad, Hercules, CA, USA) using the same five MS markers than those previously described in the Promega^®^ Analysis system (*BAT25*, *BAT26*, *Mono27*, *NR21*, *NR24*) (Table 2) [78]. This proof-of-concept study demonstrated a high concordance with conventional methods in endometrial (100% agreement, 15/15) and colorectal cancer samples (100% agreement, 15/15). Another group developed a drop-off droplet-digital PCR approach based on the analysis of BAT-26, ACVR2A, and DEFB105A/B markers into three distinct assays [80]. The drop-off ddPCR strategy presents the advantages of screening all variants in a hotspot region. It consists in the use of two TaqMan probes targeting the same amplicon: the VIC-labelled reference probe hybridizes to a nonmutated region while the FAM-labelled drop-off probe is complementary to the wild-type (WT) sequence in a frequently mutated region. In the presence of wild-type alleles, a double signal (VIC+/FAM+) was obtained. In the presence of even a single nucleotide mutation at this site, a loss of FAM signal was observed while maintaining a VIC signal (FAM−/VIC+). The limit of detection of the drop-off MSI-ddPCR was shown as low as 0.1% mutant allele frequency. This approach appears reliable in ascertaining the MSI phenotype in CRC samples (100% overall agreement) while less informative in non-colorectal cancer samples (93% overall agreement). Both MSI-ddPCR approaches appear as potential fast and affordable large-scale tools to screen MSI in multiple samples in one assay. Nevertheless, the analysis of the ddPCR raw data is currently not standardized and hence requires skilled molecular biologists for MSI interpretation.

### 3.3. NGS-Based Approaches and Computational Tools for MSI Diagnosis

Next-Generation Sequencing (NGS) currently represents a widely used technology that facilitates personalized cancer therapy through the research of numerous actionable alterations in a single assay. Recently, NGS has been adapted for the focused purpose of MSI testing. Some groups proposed NGS panels that integrate the specific detection of MSI [81,82,83]. As an example, the MSIPlus amplicon-based approach was designed for the detection of hotspots mutations in the *KRAS*, *NRAS*, and *BRAF* genes and instability in 17 microsatellite loci (including *BAT-25*, *BAT-26*, *NR-21*, *NR-24*, *MONO-27*, and *HSP110(T17)*) in colorectal cancer samples [81].

The analysis of long mononucleotide repeats (>15 bp in length) has long been preferred for MSI determination considering their high level instability in tumours [84]. However, such repeats are more prone to PCR and sequencing errors. The selection of short repeats with length ranging from 7 to 12 bp have been recently shown as an alternative for MSI classification based on the allelic distribution of mutant reads [85,86]. Moreover, shorter microsatellites were shown more monomorphic than longer ones making matched normal tissues unnecessary for the analysis. Gallon et al. optimized the technique by using single-molecule molecular inversion probes (smMIP)-based sequencing approach to detect low-frequency mutant sequences. A panel composed of only six short repeat markers was sufficient to attain 100% accuracy for MSI typing compared to conventional MSI-PCR [85].

The MSI status could also be inferred using NGS data from whole-genome, whole-exome, gene targeted, or RNA sequencing that were not originally developed for MSI diagnosis [62]. During the process of sequencing, numerous MS loci are incidentally captured along with regions of interest and can be easily identified using web-based tools such as MISA (MicroSAtellite identification tool), GMATo (Genome-wide Microsatellite Analyzing Tool), or PolyMorphPredict [26,87,88,89]. Different MSI computational tools have been developed for MSI diagnosis by exploiting existing NGS data (Table 3). Some computational approaches (such as the MSI-Seq Index, the MSIseq or the MSIPred classifier) diagnose MSI by examining the mutation load in all sequences and/or the insertions-deletions burden in microsatellite regions [90,91,92]. Others (such as mSINGS, MSIsensor or MANTIS) compare the distribution of the allele lengths at MS regions between tumour and normal samples for MSI interpretation [93,94,95].

The advantage of NGS methods over PCR-based assays is that they provide the MSI status at the same time as other relevant cancer-related alterations. This one-step approach is of particular interest for cancer types with low MSI frequency for which MSI-specific testing is not systematically performed. The widespread deployment of NGS notably highlighted the fact that the MSI status is much more prevalent than previously thought [96]. Moreover, NGS approaches can interrogate many more informative MS loci compared to the MSI-PCR assays. Considering that there is significant differences in MSI patterns across cancer types (specific MS loci unstable in a defined tumour location) [96,97], NGS could thus represent a more sensitive option when applied to cancers other than the well-studied colorectal, endometrial, and gastric tumours [98]. The analysis of multiple MS loci by NGS could also represent an option to retrieve samples for which historical techniques gave non-contributive or doubtful results. The use of NGS for MSI typing is however limited by its cost, high technical complexity, and long turnaround time and are likely to be limited to cases that require a comprehensive genomic profiling including both the MSI diagnosis and the genotyping of other genes of interest.

## 4. A Step Forward towards MSI Testing in Liquid Biopsy?

Tumour biopsy represents the reference source of material for MSI determination; however, its clinical use has several limitations. First, tissue material is sometimes inaccessible due to tumour location and results from an invasive procedure associated with potential surgical complications [104]. Second, it only gives a snapshot of the tumour diversity and may not fully capture the complexity of the disease [105]. In some rare cases of sporadic MSI/dMMR tumours, intra- and inter-tumour heterogeneity may arise as a consequence of a late emergence of MMR defects in the tumour development [106]. The characterization of MSI/dMMR status based on a single tissue biopsy could thus lead to MSI misclassification in such cases.

Liquid biopsy (LB), which refers to the analysis of circulating tumour DNA (ctDNA) shed into the body fluids (plasma, urine, saliva, …) by tumour cells, has emerged last few years as a promising surrogate for tumour biopsy. LB is presented as a minimally-invasive and easily-repeatable tool, which overcomes the issue of spatial and temporal heterogeneity and allows the longitudinal monitoring of the disease through iterative sampling [105]. LB has already been applied to detect various cancer-related alterations, including single nucleotide variations, insertion-deletion events, copy number variations, gene fusions or DNA methylation profiles [107]. Numerous studies have revealed the clinical potential of LB in establishing tumour molecular diagnostics, monitoring the treatment response, assessing the minimal residual disease, detecting early tumour relapse as well as tracking secondary resistance mechanisms [105]. LB notably revolutionized the management of patients with non–small cell lung cancers since its approval by the European Medicine agency (EMA) and the FDA for molecular profiling when tumour sampling was not possible or provided non-contributive results [108,109,110]. To date, less is known about its utility in determining MSI status.

## 5. Liquid Biopsy Technologies for MSI Detection

Considering the current trend toward tumour molecular diagnostics based on ctDNA analysis and the multiple stakes of MSI on cancer management, there is a growing need to develop novel approaches for MSI diagnosis on blood samples (bMSI). One of the major technical challenges for bMSI determination is that it requires highly sensitive methods due to the highly fragmented nature of ctDNA and the small fraction of ctDNA among total cfDNA in the body fluids (as low as 0.01% in early stages of cancers) [111]. In the last few years, significant progress has been made in improving the resolution of existing tissue-based approaches and adapting them for liquid biopsy purpose.

### 5.1. Methods for Enrichment of MSI Sequences in cfDNA

In cfDNA, altered microsatellite sequences can be missed due to their high contamination with germline DNA molecules and the presence of stutter bands at homopolymer regions when CE or HRM is employed for end-point detection. The enrichment of unstable microsatellites was proposed as an option prior to PCR-based amplification to improve the detection of low-frequency mutant alleles. The NaMe-PrO (nuclease-assisted minor-allele enrichment with probe-overlap) approach consists of overlapping oligonucleotide probes and double-strand-specific nucleases, which specifically eliminate long unaltered homopolymer regions while sparing those harbouring indels. NaMe-PrO combined with CE or HRM attained a limit of detection of 0.01% mutant microsatellite allele frequencies [112,113]. However, this strategy is limited by the number of MS that could be targeted.

In order to efficiently capture low fraction cfDNA present in plasma samples, Yu et al. developed an inter-Alu-PCR-NGS approach that combines a fast and easy-to perform PCR assay with a NGS-based broad molecular profiling [114]. Alu sequences are short interspersed nuclear elements that accounts for almost 11% of the human genome [115]. Alu elements harbour a 3′ polyA tail forming a microsatellite-like sequence with variable length. Alu primers target regions away from the alu sequence and amplify homopolymer sequence between two neighbor alu elements, enabling the enrichment of multiple MS loci in meantime. As primers are composed of NGS adapter sequences, the library construction can be performed directly after inter-Alu-PCR. A custom MSI-tracer algorithm finally compares the homopolymer read length between cfDNA from cancer patients and healthy volunteers. Using as little as 0.1–1 ng of cfDNA, Inter-Alu-PCR-NGS was able to distinguish plasma DNA from patients with and without microsatellite instability.

### 5.2. ddPCR Assays as a Viable Option for bMSI Determination

Silveira et al. evaluated the analytical performance of the previously described 3-marker (BAT-26, ACVR2A, DEFB105A/B) drop-off ddPCR approach on blood samples from patients with advanced or metastatic CRC and endometrial cancers [80]. Using tissue MSI status as the gold standard, the MSI-ddPCR method attained 100% sensitivity and specificity. Moreover, it provided absolute quantification of the MSI sequences, making this approach compatible with longitudinal ctDNA monitoring.

### 5.3. Improvement of Tissue-Based NGS Approaches and Computational Algorithms for bMSI Determination

The widespread employment of ctDNA-based NGS approaches is limited by prevailing biological and technological hurdles. The low abundance of ctDNA in body fluids along with technical artifacts generated during library preparation, amplification, or sequencing lessen the analytical sensitivity of these methods [116]. In the last few years, advances in NGS technology have been made in order to optimize the detection of low-frequency ctDNA. Such technical improvements were notably employed in recent integrated cfDNA-based pan-cancer NGS approaches designed for bMSI ascertainment [117,118,119] (Table 4). The Guardant360^®^ CDx (Guardant Health, Redwood city, CA, USA) and FoundationOne^®^ Liquid CDx (Foundation Medicine, Cambridge, MA, USA) are commercial FDA-approved blood-based companion diagnostics that have been adapted for specific bMSI determination. The Guardant360^®^ CDx, FoundationOne^®^ Liquid CDx, OncoLBx and Georgiadis approaches all employ hybrid-capture enrichment of target regions and rely on molecular barcoding to help filter false positive events arising due to technical PCR errors. They also integrate in silico error correction approaches in order to reduce the background noise and accurately recover true insertion-deletion events in MS regions. Due to the fragmented nature of ctDNA, Willis et al. showed that some MS loci traditionally used for tissue-based MSI testing are not suitable for ctDNA-based NGS approaches due to low coverage or background noise among these regions [118]. In Guardant360^®^ panel, they selected limited but highly informative MS loci based on their coverage and noise profiles in order to improve molecular capture and mapping efficiency. Along with bMSI status, Georgiadis and FoundationOne^®^ Liquid CDx methods offer the possibility to determine blood based tumour mutation burden (bTMB), a complementary biomarker that help inform ICI treatment. For all panels described, a minimum of 5–30 ng cfNDA input was required. To note, some groups implemented specific bMSI computational algorithms for NGS data in the context of liquid biopsy analysis, enabling the detection of very low fraction of ctDNA (0.05–0.5%) (Table 5).

## 6. Perspectives of the Applications of Liquid Biopsy in MSI Testing

A good overall agreement has been observed between conventional MSI tissue-based testing and newly developed ctDNA-based approaches [80,117,118]. This suggests that ctDNA-based MSI diagnosis could be performed as part of routine clinical practice to stratify patients with better prognosis that are likely to benefit from ICI, when tissue specimens are unavailable or scarce [117,118,122]. Through its minimally-invasive nature, liquid biopsy can be serially repeated in order to ensure real-time disease monitoring based on ctDNA kinetics. Changes in bMSI levels during ICI treatment correlated well with those of other ctDNA markers and reliably reflected tumour response to treatment [80]. In a limited subset of patients under ICI treatment, the residual bMSI allele burden was found inversely correlated with the overall and progression-free survival and allowed an earlier prediction of tumour response compared to conventional radiographic imaging [117]. Longitudinal ctDNA analysis also allowed the detection of somatic MSI acquisition that can appear during cancer evolution in patients initially diagnosed with MSS tumours [127]. To our knowledge, only few cases were demonstrated to acquire MSI phenotype during the disease course [127,128]; however, such phenomenon may have been underestimated given that most tumours are screened for MSI only at the time of diagnosis in routine practice. The interest of such acquired MSI phenotype to guide treatment decision remains elusive and needs to be demonstrated by further studies. The use of such strategy based on the analysis of serial plasma samples, however, dramatically increases the cost of MSI testing. In this context, cost-effectiveness analyses should be performed prior to its implementation in clinical practice.

## 7. Conclusions

Determination of MSI status in cancers is of particular clinical importance considering its diagnostic, prognostic, and therapeutic significance. IHC and pentaplex PCR account for MSI/dMMR standard reference methods on tumour tissue specimens. However, other approaches (such as custom NGS approaches or computational algorithms for NGS data, real-time PCR or ddPCR assays using custom MS panels) have emerged and progressively entered into clinical practice. Given the multiple methods currently available, the approach to be used should be chosen considering the cancer type, the preanalytical conditions, the lab resources and technical expertise, and the availability of paired normal tissues.

In the last few years, liquid biopsy led to a major paradigm shift in oncology, providing a viable surrogate to tissue biopsy for molecular investigations. As other ctDNA markers, bMSI can be detected in body fluids and contributes to predict treatment efficacy and follow disease evolution over time. The use of bMSI-based strategies has been initially confounded by lack of sensitivity; however, recent technological advances in the field showed potential in reducing background noises and enhancing detection efficiency. Further translational studies are needed to confirm the clinical utility of bMSI-based approaches and delineate their potentials applications in routine practice.

## Figures and Tables

**Table 1:** Characteristics of the standard reference methods for MSI/dMMR determination.

Methods	Markers Analyzed	Interpretation of the Results	Advantages	Limitations
IHC	A set of 2 (MSH6/PMS2) or 4 MMR proteins (MLH1/MSH2/MSH6/PMS2) [35]	The loss of at least 1 MMR protein defines dMMR tumours	- Fast turnaround time for results (~4–6 h) - Easy to institute in all clinical laboratories - Feasible in samples with <20% neoplastic cells - Low cost - Helpful in identifying the MMR genes to investigate for mutation analysis	- Separate analyses of the four MMR proteins - Requirement for an expert pathologist to interpret the results [51] - Equivocal test results due to the heterogeneous expression of MMR proteins - False-positive results: artificial loss of expression due to pre-analytic issues or lack of technical calibration [49] - Rare false-negative results: no apparent loss of expression due to missense mutations in the MMR genes with intact immunoreactivity in 10% of cases [49]
Pentaplex MSI-PCR followed by capillary electrophoresis	5 mononucleotide and quasi-monomorphic MS markers (including BAT-25 and BAT-26) [38,39,40]	Tumours harbouring ≥ 40% of MS markers (≥2 out of the 5 mononucleotide MS markers) unstable are considered MSI-H.	- Multiplexed - Highly reproducible - Fast turnaround time for results (<5 h) - Low cost	- No indication about the MMR genes to investigate - Requirement for samples with at least 20% neoplastic cells - Rare false-positive results due to microsatellite polymorphisms [50] - Informative only for few cancer types due to the limited number of targets

Abbreviations: dMMR: deficient DNA Mismatch Repair; IHC: imumunohistochemistry; MLH1: MutL Homolog human 1; MMR: DNA Mismatch Repair; MS: microsatellites; MSH2: MutS Homolog human 2; MSH6: MutS Homolog human 6; MSI-H: Microsatellite instability with high confidence; PMS2: Postmeiotic Segregation Increased 2.

**Table 2:** Examples of emerging tissue-based methods for MSI detection.

Methods	Markers Analyzed	Interpretation of the Results	Advantages	Limitations
*LMR-MSI PCR* [58,59]	8 MS markers including 4 traditional markers (*BAT25*, *BAT26*, *Mono27*, *NR21* and *NR24*) and 4 LMR markers (*BAT-52*, *BAT-56*, *BAT-59* and *BAT-60*) [58]	Tumours harbouring ≥ 2 MS markers unstable are considered MSI-H. Tumours harbouring 1 MS marker unstable are defined MSI-L [58]	- Better sensitivity than the pentaplex PCR [58,59] - Multiplexed - Fast turnaround time for results (<5 h) - Low cost	- No indication about the MMR genes to investigate - Designed for colorectal cancer samples; no information about its performance in non-colorectal cancers - Need for matched normal tissue sample
*HSP110 (T17)* PCR or E-ice-COLD-*PCR* [68,69,71]	*HSP110 (T17)* quasi-monomorphic marker	Allelic mutations in the *HSP110 (T17)* gene define MSI tumours	- Require the analysis of a unique marker - Highly reproducible - Better sensitivity than the pentaplex PCR [68] - Fast turnaround time for results (<5 h) - Low cost - No need for matched normal tissue sample	- No indication about the MMR genes to investigate - Designed for colorectal cancer samples; no information about its performance in non-colorectal cancers
Idylla^®^ MSI test [78,79]	7 monomorphic MS markers (*ACVR2A*, *BTBD7*, *DIDO1*, *MRE11*, *RYR3*, *SEC31A*, *SULF2*) [74]	Tumours harbouring ≥ 2 out of the 7 MS markers unstable are considered MSI-H.	- Multiplexed - Highly reproducible - Minimal hands-on-time (~5 min) [78] - Fast turnaround time for results (~2.5 h) [78] - Low cost - No need for previous DNA extraction - No need for matched normal tissue sample	- No indication about the MMR genes to investigate - Requirement for samples with at least 20% neoplastic cells [78] - Initially designed for colorectal cancer samples; performance of the Idylla^®^ assay confirmed in gastro-intestinal and endometrial cancers [78,79]; need further evaluations in other cancer types
Bio-Rad^®^ pentaplexddPCR [78]	5 quasi-monomorphic MS markers (*BAT25*, *BAT26*, *Mono27*, *NR21* and *NR24*)	Tumours with at least 2 markers out of 5 (≥40% of MS markers) unstable are defined as having MSI-H.	- Fast turnaround time for results *(*<5 h) - Low cost - No need for matched normal tissue sample	- No indication about the MMR genes to investigate - Lack of standardization for data interpretation - Informative only for few cancer types due to the limited number of targets
Drop-off ddPCR [80]	3 MS markers (BAT-26, ACVR2A, DEFB105A/B)	Tumours with at least 2 markers out of 3 unstable are defined as having MSI-H.	- Fast turnaround time for results *(*<5 h) - Low cost - Highly informative for MSI in CRC cancers (100% overall concordance) - limit of detection <0.1% mutant allele frequency - No need for matched normal tissue sample	- No indication about the MMR genes to investigate - Lack of standardization for data interpretation - Less informative for MSI in non-colorectal cancer types (93% overall concordance)

Abbreviations: ACVR2A: activin A receptor type 2A; BTBD7: BTB domain containing 7; CRC: colorectal; ddPCR: droplet digital PCR; DEFB105A/B: defensin beta 105A/B; DIDO1: Death Inducer-Obliterator 1; E-ice-COLD PCR: Enhanced Improved and Complete Enrichment CO-amplification at Lower Denaturation temperature PCR; LMR: long mononucleotide repeats; MMR: DNA Mismatch Repair; MRE11: meiotic recombination 11; MS: microsatellites; MSI: Microsatellite instability; MSI-H: Microsatellite instability with high confidence; MSI-L: Microsatellite instability with low confidence; RYR3: Ryanodine receptor 3; EC31A: SEC31 Homolog A, COPII Coat Complex Component; SULF2: Sulfatase 2.

**Table 3:** Examples of NGS-based computational tools for MSI detection.

Strategy	Computational Tool	Samples to Be Analyzed	Principle of the Algorithm	Scoring Interpretation (Default Cut-Off Thresholds)
Mutation burden	MSIpred [90]	Tumour	MSI prediction based on 22 features characterizing tumour mutational load.	Binary non-MSI-H/MSI-H classification (Interpretation using a support vector machine classifier)
	MSIseq [91]	Tumour	MSI prediction based on 9 features characterizing tumour mutational load.	Binary non-MSI-H/MSI-H classification (Interpretation using a decision tree classifier)
	MSIseq index [92]	Tumour	MSI status is determined from RNA sequencing data. MSI prediction is based on the proportion of insertions in MS regions over all insertions (PI) and the proportion of deletions in MS over all deletions (PD).	Binary MSS/MSI classification (MSI if ratio [PI/PD] < 0.9)
	Nowak [99]	Tumour	MSI prediction based on total mutation load and indels burden in MS regions.	Binary MSS/MSI classification (MSI if >40 total mutations/Mb or 5 indels in MS loci/Mb)
	preMSIm [100]	Tumour	MSI prediction based on the expression profile of 15 gene signatures.	Binary non-MSI-H/MSI-H classification (Interpretation using *k*-Nearest Neighbours classifier)
	MIRMMR [101]	Tumour	MSI prediction based on methylation and mutation data from MMR pathway genes	Binary non-MSI-H/MSI-H classification (MSI if MIRMMR scores > 0.1922)
	Cortes-Ciriano method [97]	Tumour vs. paired normal samples	Kolmogorov-Smirnov test to evaluate the difference in read length distribution at each locus.	Binary MSS/MSI classification (Interpretation based on a random forest approach)
Allele length distribution at MS loci	MOSAIC [96]	Tumour vs. paired normal samples	Average gain in the number of microsatellite alleles and locus instability	Binary MSS/MSI-H classification (Interpretation using a decision tree classifier)
	MANTIS [93]	Tumour vs. paired normal samples	Difference in read length distribution at each locus is established, then an average difference score across all MS locus is calculated.	Binary MSS/MSI classification (MSI if average difference score > 0.4)
	NovoPM-MSI [102]	Tumour vs. paired normal samples	Mann-Whitney U test to evaluate the difference in read length distribution at each locus.	Binary MSS/MSI classification (MSI if >20% unstable loci)
	MSI sensor [94]	Tumour vs. paired normal samples	Chi2 test to evaluate the difference in read length distribution at each locus.	Binary MSS/MSI classification (MSI if >3.5% unstable loci)
	MSI sensor pro [103]	Tumour vs. baseline	Quantification of polymerase slippage events. The selection of discriminative MS sites obviates the need for normal tissue samples.	Binary MSS/MSI classification (Interpretation based on a multinomial distribution model)
	mSINGS [95]	Tumour vs. baseline	Z-test to evaluate the difference in read length distribution at each locus.	Binary MSS/MSI classification (MSI if >20% unstable loci)
	MSI-ColonCore algorithm [83]	Tumour vs. baseline	Z-test to evaluate the difference in read length distribution at each locus.	Ternary MSS/MSI-L/MSI-H classification (MSI-H if >40% unstable loci MSI-L if 15–40% unstable loci)

Abbreviations: indels: insertions-deletions; MANTIS: Microsatellite Analysis for Normal-Tumor InStability; MS: microsatellite; MSI-H: microsatellite instability with high confidence; MSI-L: microsatellite instability with low confidence; mSINGS: Microsatellite Instability By Next-Generation Sequencing; MOSAIC: MicrOSAtellite Instability Classifier; MSS: microsatellite stability; preMSIm: Predicting MSI from mRna.

**Table 4:** Examples of integrated cfDNA-based pan-cancer NGS approaches for bMSI determination.

NGS Approach	Panel	Enrichment Method	cfDNA Input	Molecular Barcoding	bTMB Determination	Number of MS Loci Assessed	Bioinformatic Tools	Scoring Interpretation (thresholds)	Analytical Performance
Georgiadis method [117]	58-gene panel	capture	5–250 ng	Yes	Yes	*5 MS loci* (*BAT25*, *BAT26*, *MONO27*, *NR21* and *NR24*)	Multifactorial error correction approach and digital peak finding algorithm	Binary bMSS/bMSI-H classification bMSI if bMSIscore ≥ 20%	78% sensitivity (18/23) and 100% specificity (6/6) Gold standard: NGS tissue-testing
OncoLBx [120]	75-gene panel	capture	20–30 ng	Yes	No	5 MS loci (*BAT25*, *BAT26*, *MONO27*, *NR21* and *NR24*)	SMSEQ error correction	Binary bMSS/bMSI-L/bMSI-H classification bMSI-H if ≥40% unstable loci bMSI-L if 20–40 unstable loci	LOD: 2% tumour fraction
Guardant360^®^ CDx [118,121,122]	74-gene panel	capture	5–30 ng	Yes	No	90 MS loci	Digital Sequencing error correction approach	Binary bMSS/bMSI-H classification (bMSI-score >6 unstable loci)	87% sensitivity (71/82) and 99.5% specificity (863/867) Gold standard: IHC, PCR or NGS tissue-testing LOD: 0.09–0.1% ctDNA content
FoundationOne^®^ Liquid CDx [119]	324-gene panel	capture	~20–30 ng	Yes	Yes	~2000 MS loci	For a given MS locus, the homopolymer length is compared to an average length (calculated on more than 3000 clinical samples). An MSI indicator is calculated based on the proportion of unstable loci	Binary bMSS/bMSI-H classification >bMSI-H if >0.5% unstable loci	LOD: 0.8% unstable loci

Abbreviations: bMSI-H: microsatellite instability with high confidence based on blood testing; bMSI-L: microsatellite instability with low confidence based on blood testing; bMSS: microsatellite stability based on blood testing; bTMB: blood-based tumour mutation burden; cfDNA: cell-free DNA; ctDNA: circulating tumour DNA; LOD: limit of detection; MS: microsatellite; NGS: next-generation sequencing; SMSEQ: single molecule sequencing. Based on the literature, cfDNA concentration in plasma ranges from 1.8 to 44 ng/mL with an average of 30 ng/mL [123].

**Table 5:** Examples of NGS-based computational tools for bMSI detection.

Computational Tool	Principle of the Algorithm	Scoring Interpretation (Default Cut-Off Thresholds)	Analytical Performance
bMSISEA [124]	Establishment of a baseline MS signature based on the analysis of white blood cells from 100 MSS CRC patients. Evaluation of the enrichment of the MSI pattern at each MS locus in tested plasma sample compared to baseline.	Binary bMSS/bMSI-H classification bMSI-H if bMSI score > 15	94.1% sensitivity (16/17) 100% specificity (27/27) for samples with ctDNA content > 0.4%
MSIsensor-ct [125]	Use of 1476 site-classifiers obtained by a machine learning model based on the read length distribution at MS loci in solid tumour NGS data. No need for preconstructed baseline control.	Binary bMSS/bMSI classification bMSI if bMSIscore ≥ 20%	100% sensitivity and specificity in 39 samples and 17 simulated datasets. LOD: 0.05% ctDNA content with at least 3000× read depth.
Wang method [126]	Difference in read length distribution at each locus. A bMSI score is established as the proportion of unstable loci among the selected 100 MS loci.	Binary bMSS/bMSI classification bMSI-H if bMSI score ≥ 0.2	82.5% sensitivity (33/40) 96.2% specificity (201/209) 94% overall concordance (234/249) Gold standard: tissue-based pentaplex PCR LOD: 0.5% at 30 ng of ctDNA input

Abbreviations: bMSI: microsatellite instability based on blood testing; bMSI-H: microsatellite instability with high confidence based on blood testing; bMSISEA: blood MSI signature enrichment analysis; bMSS: microsatellite stability based on blood testing; ctDNA: circulating tumour DNA; LOD: limit of detection; MS: microsatellites, NGS: next-generation sequencing; bTMB: blood-based tumour mutation burden.

## Data Availability

Not applicable.

## Abbreviations

bTMB	blood based tumour mutation burden
DHPLC	Denaturing High-Performance Liquid Chromatography
dMMR	deficient MisMatch Repair
EPCAM	EPithelial Cell Adhesion Molecule
FDA	Food and Drug Administration
HRM	High Resolution Melting
HSP110	Heat Shock Protein 110
indels	insertions-deletions
LS	Lynch Syndrome
MANTIS	Microsatellite Analysis for Normal Tumor InStability
MIMMR	Microsatellite Instability Regression using Methylation and Mutations in R
MLH1	MutL Homolog human 1
MMR	mismatch repair
MOSAIC	MicrOSAtellite Instability Classifier
MS	Microsatellites
MSH2	MutS Homolog human 2
MSH6	MutS Homolog human 6
MSI	microsatellite instability
mSINGS	microSatellite Instability by Next-Generation Sequencing
MSS	Microsatellite Stability
NaMe-PrO	nuclease-assisted minor-allele enrichment with probe-overlap
NGS	Next-Generation Sequencing
PMS1	Postmeiotic Segregation Increased 1
PMS2	Postmeiotic Segregation Increased 2
preMSIm	Predicting MSI from mRna
